# Cunningham's Manual of Practical Anatomy

**Published:** 1915-06

**Authors:** 


**Cunningham's Manual of Practical Anatomy.** Sixth Edition. Revised and Edited by Arthur Robinson. Vol. II. Pp. xxx., 636. London: Henry Frowde and Hodder and Stoughton. 1914. Price 10s. 6d. net.

The rapid appearance of the sixth edition of this manual testifies to its well-deserved popularity. There are a number of new illustrations of varying merit, but the most important new feature lies in the number of reproductions of radiographs of the head and neck and thorax. Radiography has become such an important factor in diagnosis in medicine and surgery, that it becomes necessary that the student should be made familiar with the normal radiographic appearances, for otherwise he can scarcely be expected to diagnose abnormal conditions when engaged in clinical work.

The section on the brain has been very largely re-written, and also contains most of the new illustrations.

The new anatomical nomenclature has, fortunately for the student, not been exclusively adopted, as in nearly every case the old term is bracketed with the new.

